# Opportunities and challenges for monitoring terrestrial biodiversity in the robotics age

**DOI:** 10.1038/s41559-025-02704-9

**Published:** 2025-05-22

**Authors:** Stephen Pringle, Martin Dallimer, Mark A. Goddard, Léni K. Le Goff, Emma Hart, Simon J. Langdale, Jessica C. Fisher, Sara-Adela Abad, Marc Ancrenaz, Fabio Angeoletto, Fernando Auat Cheein, Gail E. Austen, Joseph J. Bailey, Katherine C. R. Baldock, Lindsay F. Banin, Cristina Banks-Leite, Aliyu S. Barau, Reshu Bashyal, Adam J. Bates, Jake E. Bicknell, Jon Bielby, Petra Bosilj, Emma R. Bush, Simon J. Butler, Dan Carpenter, Christopher F. Clements, Antoine Cully, Kendi F. Davies, Nicolas J. Deere, Michael Dodd, Rosie Drinkwater, Don A. Driscoll, Guillaume Dutilleux, Mads Dyrmann, David P. Edwards, Mohammad S. Farhadinia, Aisyah Faruk, Richard Field, Robert J. Fletcher, Chris W. Foster, Richard Fox, Richard M. Francksen, Aldina M. A. Franco, Alison M. Gainsbury, Charlie J. Gardner, Ioanna Giorgi, Richard A. Griffiths, Salua Hamaza, Marc Hanheide, Matt W. Hayward, Marcus Hedblom, Thorunn Helgason, Sui P. Heon, Kevin A. Hughes, Edmund R. Hunt, Daniel J. Ingram, George Jackson-Mills, Kelly Jowett, Timothy H. Keitt, Laura N. Kloepper, Stephanie Kramer-Schadt, Jim Labisko, Frédéric Labrosse, Jenna Lawson, Nicolas Lecomte, Ricardo F. de Lima, Nick A. Littlewood, Harry H. Marshall, Giovanni L. Masala, Lindsay C. Maskell, Eleni Matechou, Barbara Mazzolai, Alistair McConnell, Brett A. Melbourne, Aslan Miriyev, Eric Djomo Nana, Alessandro Ossola, Sarah Papworth, Catherine L. Parr, Ana Payo-Payo, Gad Perry, Nathalie Pettorelli, Rajeev Pillay, Simon G. Potts, Miranda T. Prendergast-Miller, Lan Qie, Persie Rolley-Parnell, Stephen J. Rossiter, Marcus Rowcliffe, Heather Rumble, Jon P. Sadler, Christopher J. Sandom, Asiem Sanyal, Franziska Schrodt, Sarab S. Sethi, Adi Shabrani, Robert Siddall, Simón C. Smith, Robbert P. H. Snep, Carl D. Soulsbury, Margaret C. Stanley, Philip A. Stephens, P. J. Stephenson, Matthew J. Struebig, Matthew Studley, Martin Svátek, Gilbert Tang, Nicholas K. Taylor, Kate D. L. Umbers, Robert J. Ward, Patrick J. C. White, Mark J. Whittingham, Serge Wich, Christopher D. Williams, Ibrahim B. Yakubu, Natalie Yoh, Syed A. R. Zaidi, Anna Zmarz, Joeri A. Zwerts, Zoe G. Davies

**Affiliations:** 1 Durrell Institute of Conservation and Ecology (DICE), School of Natural Sciences, University of Kent, Canterbury, UK; 2 Centre for Environmental Policy, Imperial College London, London, UK; 3 Department of Geography and Environmental Sciences, Northumbria University, Newcastle upon Tyne, UK; 4 School of Computing, Engineering and the Built Environment, Edinburgh Napier University, Edinburgh, UK; 5 Synthotech Ltd, Milner Court, Hornbeam Square, Harrogate, UK; 6 Mechanical Engineering Department, University College London, London, UK; 7 HUTAN, SWD, Kota Kinabalu, Malaysia; 8 Programa de Pós-Graduação em Gestão e Tecnologia Ambiental da Universidade Federal de Rondonópolis, Rondonópolis, Brasil; 9 Department of Engineering, Harper Adams University, Newport, UK; 10 Applied Ecology Research Group, School of Life Sciences, Anglia Ruskin University, Cambridge, UK; 11 Centre for Ecology and Hydrology, Penicuik, UK; 12 Department of Life Sciences, Imperial College London, Silwood Park Campus, Ascot, UK; 13 Department of Urban and Regional Planning, Faculty of Earth and Environmental Sciences, Bayero University, Kano, Nigeria; 14 Greenhood Nepal, Kathmandu, Nepal; 15 Animal, Rural & Environmental Sciences, Nottingham Trent University, Nottinghamshire, UK; 16 School of Biological and Environmental Sciences, Liverpool John Moores University, Liverpool, UK; 17 Lincoln Centre for Autonomous Systems, University of Lincoln, Lincoln, UK; 18 Royal Botanic Garden Edinburgh, Edinburgh, UK; 19 School of Biological Sciences, University of East Anglia, Norwich Research Park, Norwich, UK; 20 Digital Ecology Limited, Bristol, UK; 21 School of Biological Sciences, University of Bristol, Bristol, UK; 22 Department of Computing, Imperial College London, London, UK; 23 Department of Ecology and Evolutionary Biology, University of Colorado, Boulder, CO USA; 24 Faculty of Science Technology, Engineering and Mathematics (STEM), The Open University, Walton Hall, Milton Keynes, UK; 25 Palaeogenomics Group, Faculty of Veterinary Medicine, Ludwig Maximilian University Munich, Munich, Germany; 26 School of Life and Environmental Sciences, Deakin University, Melbourne Burwood Campus, Australia; 27 Acoustics Group, Department of Electronic Systems, Norwegian University of Science and Technology, Trondheim, Norway; 28 AI Lab ApS, Maden 3, Aarhus V, Denmark; 29 Department of Plant Sciences and Centre for Global Wood Security, University of Cambridge, Cambridge, UK; 30 Royal Botanic Gardens Kew, Millennium Seed Bank, Wakehurst Place, Ardingly, UK; 31 School of Geography, University of Nottingham, Nottingham, UK; 32 Department of Zoology, University of Cambridge, Cambridge, UK; 33 School of Biological Sciences, University of Reading, Reading, UK; 34 Butterfly Conservation, Manor Yard, East Lulworth, UK; 35 Institute of Science and Environment, University of Cumbria, Carlisle, UK; 36 School of Environmental Sciences, University of East Anglia, Norwich, UK; 37 Department of Integrative Biology, University of South Florida, St Petersburg, FL USA; 38 School of Computing, University of Kent, Canterbury, UK; 39 Biomorphic Intelligence Lab, Department of Control & Operations, Faculty of Aerospace Engineering, TU Delft, Delft, The Netherlands; 40 Centre for Conservation Science, University of Newcastle, Callaghan, Newcastle, New South Wales Australia; 41 Department of Urban and Rural Development, Swedish University of Agricultural Sciences, Uppsala, Sweden; 42 School of Biological Sciences, University of Edinburgh, Edinburgh, UK; 43 Southeast Asia Rainforest Research Partnerships, Kota Kinabalu, Malaysia; 44 British Antarctic Survey, Natural Environment Research Council, High Cross, Cambridge, UK; 45 School of Engineering Mathematics and Technology, University of Bristol, Bristol, UK; 46 Institute of Design, Robotics, and Optimisation (iDRO), School of Mechanical Engineering, University of Leeds, Leeds, UK; 47 Rothamsted Research, Harpenden, UK; 48 Department of Integrative Biology, University of Texas at Austin, Austin, TX USA; 49 Department of Biological Sciences, University of New Hampshire, Durham, NH USA; 50 Department of Ecological Dynamics, Leibniz Institute for Zoo and Wildlife Research IZW, Berlin, Germany; 51 Research Department of Genetics, Evolution and Environment, University College London, London, UK; 52 Intelligent Robotics Group, Department of Computer Science, Aberystwyth University, Aberystwyth, UK; 53 UK Centre for Ecology and Hydrology (CEH), Wallingford, UK; 54 Department of Biology and Centre d’Études Nordiques, University of Moncton, Moncton, Canada; 55 Center for Ecology, Evolution and Environmental Changes, Departamento de Biologia Animal, Faculdade de Ciências da Universidade de Lisboa, Lisboa, Portugal; 56 Scotland’s Rural College (SRUC), Aberdeen, UK; 57 RSPB Centre for Conservation Science, Cambridge, UK; 58 UKCEH, Lancaster Environment Centre, Bailrigg, UK; 59 School of Engineering, Mathematics and Physics, University of Kent, Canterbury, UK; 60 Istituto Italiano di Tecnologia (IIT), Bioinspired Soft Robotics Laboratory, Genoa, Italy; 61 School of Mathematical and Computer Sciences, Heriot-Watt University, Edinburgh, UK; 62 Physical AI (PAI) Laboratory, Department of Mechanical Engineering, Ben-Gurion University of the Negev, Beer-Sheva, Israel; 63 Interdisciplinary Centre for Conservation Biology, Department of Biology, University of Oxford, Oxford, UK; 64 Department of Plant Sciences, University of California Davis, Davis, CA USA; 65 Department of Biological Sciences, Royal Holloway University of London, Egham, UK; 66 School of Environmental Sciences, University of Liverpool, Liverpool, UK; 67 Departamento de Biodiversidad, Ecología y Evolución, Facultad de Ciencias Biológicas, Universidad Complutense, Madrid, Spain; 68 Department of Environmental Science and Policy, College of Science, George Mason University, Fairfax, VA USA; 69 Institute of Zoology, Zoological Society of London, Regent’s Park, London, UK; 70 Natural Resources and Environmental Studies Institute, University of Northern British Columbia, Prince George, British Columbia Canada; 71 Centre for Agri-Environmental Research (CAER), School of Agriculture, Policy and Development, Reading University, Reading, UK; 72 School of Natural Sciences, University of Lincoln, Lincoln, UK; 73 Insect Robotics Group, School of Informatics, University of Edinburgh, Edinburgh, UK; 74 School of Biological and Behavioural Sciences, Queen Mary University of London, London, UK; 75 Centre for Sustainable Planning and Environments, University of the West of England Bristol, Bristol, UK; 76 School of Geography, Earth and Environmental Sciences, University of Birmingham, Birmingham, UK; 77 School of Life Sciences, University of Sussex, Brighton, UK; 78 Fauna & Flora (International), The David Attenborough Building, Cambridge, UK; 79 Department of Life Sciences, Imperial College London, London, UK; 80 Sabah Landscape Programme, WWF-Malaysia, Kota Kinabalu, Malaysia; 81 Institute for Sustainability, School of Mechanical Engineering Sciences, University of Surrey, Guildford, UK; 82 Wageningen Environmental Research, Wageningen University & Research (WUR), Wageningen, The Netherlands; 83 School of Biological Sciences, Waipapa Taumata Rau – University of Auckland, Auckland, New Zealand; 84 Conservation Ecology Group, Department of Biosciences, Durham University, Durham, UK; 85 IUCN SSC Species Monitoring Specialist Group, Laboratory for Conservation Biology, Department of Ecology & Evolution, University of Lausanne, Lausanne, Switzerland; 86 Bristol Robotics Laboratory, UWE Bristol, Bristol, UK; 87 Department of Forest Botany, Dendrology and Geobiocoenology, Mendel University in Brno, Brno, Czech Republic; 88 Centre for Robotics and Assembly, School of Aerospace, Transport and Manufacturing (SATM), Cranfield University, Bedford, UK; 89 Edinburgh Centre for Robotics (ECR), School of Mathematical and Computer Sciences, Heriot-Watt University, Edinburgh, UK; 90 School of Science, Western Sydney University, Penrith, New South Wales Australia; 91 Amphibian and Reptile Conservation, Boscombe, UK; 92 Centre for Conservation & Restoration Science, Edinburgh Napier University, Edinburgh, UK; 93 School of Natural and Environmental Sciences, Newcastle University, Newcastle, UK; 94 Department of Environmental Management, Faculty of Earth and Environmental Sciences, Bayero University, Kano, Nigeria; 95 School of Electronic and Electrical Engineering, University of Leeds, Leeds, UK; 96 Department of Geoinformatics, Cartography and Remote Sensing, Faculty of Geography and Regional Studies, University of Warsaw, Warsaw, Poland; 97 Ecology and Biodiversity, Utrecht University, Utrecht, The Netherlands

**Keywords:** Ecosystem ecology, Conservation biology

## Abstract

With biodiversity loss escalating globally, a step change is needed in our capacity to accurately monitor species populations across ecosystems. Robotic and autonomous systems (RAS) offer technological solutions that may substantially advance terrestrial biodiversity monitoring, but this potential is yet to be considered systematically. We used a modified Delphi technique to synthesize knowledge from 98 biodiversity experts and 31 RAS experts, who identified the major methodological barriers that currently hinder monitoring, and explored the opportunities and challenges that RAS offer in overcoming these barriers. Biodiversity experts identified four barrier categories: site access, species and individual identification, data handling and storage, and power and network availability. Robotics experts highlighted technologies that could overcome these barriers and identified the developments needed to facilitate RAS-based autonomous biodiversity monitoring. Some existing RAS could be optimized relatively easily to survey species but would require development to be suitable for monitoring of more ‘difficult’ taxa and robust enough to work under uncontrolled conditions within ecosystems. Other nascent technologies (for instance, new sensors and biodegradable robots) need accelerated research. Overall, it was felt that RAS could lead to major progress in monitoring of terrestrial biodiversity by supplementing rather than supplanting existing methods. Transdisciplinarity needs to be fostered between biodiversity and RAS experts so that future ideas and technologies can be codeveloped effectively.

## Abstract (web summary)

An expert-elicitation process identifies current methodological barriers for monitoring terrestrial biodiversity, and how technological and procedural development of robotic and autonomous systems may contribute to overcoming these challenges.

## Main

To conserve biodiversity effectively, we must be able to accurately and comprehensively monitor species populations to anticipate and ameliorate declines proactively^1^. This is critical, given that recent projections suggest that up to two million species are at risk of extinction, with plants and invertebrates most at threat^2^. Indeed, conservationists need to monitor biodiversity across all ecosystems, from urban areas to inaccessible wilderness, to mitigate the drivers of species loss. These monitoring programmes need to be robust, predicting future species extinctions and ecosystem collapse well in advance of tipping points being reached.

Monitoring terrestrial biodiversity is time-consuming and expensive to replicate spatially and temporally. Many ecological relationships only become apparent following extensive surveys over broad geographic scales, often through time, which can be unfeasible using current methods^3^. Comprehensive monitoring might encompass tens, hundreds or even thousands of sites that need repeated and ideally synchronous surveying. Biodiversity monitoring also requires expertise in field observation and, for some taxa, detailed knowledge of taxonomy or the use of specialist techniques such as collection and analysis of genetic material^4^. In addition, species frequently have restricted habitat associations, meaning that the effectiveness of monitoring can be severely hampered or biased by environmental factors, including whether researchers can access sites and tolerate the conditions within them. Human surveyors can also overlook cryptic, elusive and small species^5^. Overcoming these constraints requires a step change in the methods used to monitor terrestrial species populations across all taxonomic groups.

RAS are technologies that can sense, analyse, interact with and manipulate their physical environment^6^. RAS have been developed for many applications (for instance, military applications^7^, agriculture^8^, infrastructure maintenance^9^ and surgery^10^) and in recent years have been widely adopted for monitoring of marine ecosystems^11^. The core technology underpinning current applications may also offer the potential to complement and/or extend our terrestrial biodiversity monitoring capabilities^12^. For example, an uncrewed aerial vehicle (UAV)-borne tool (https://outreachrobotics.com) suited to infrastructure inspection has been used to sample plants from inaccessible cliffs. Likewise, technology developed for inspection and maintenance of below-ground pipes and sewers (https://pipebots.ac.uk) could be used to survey species that inhabit burrows.

**Fig. 1:**
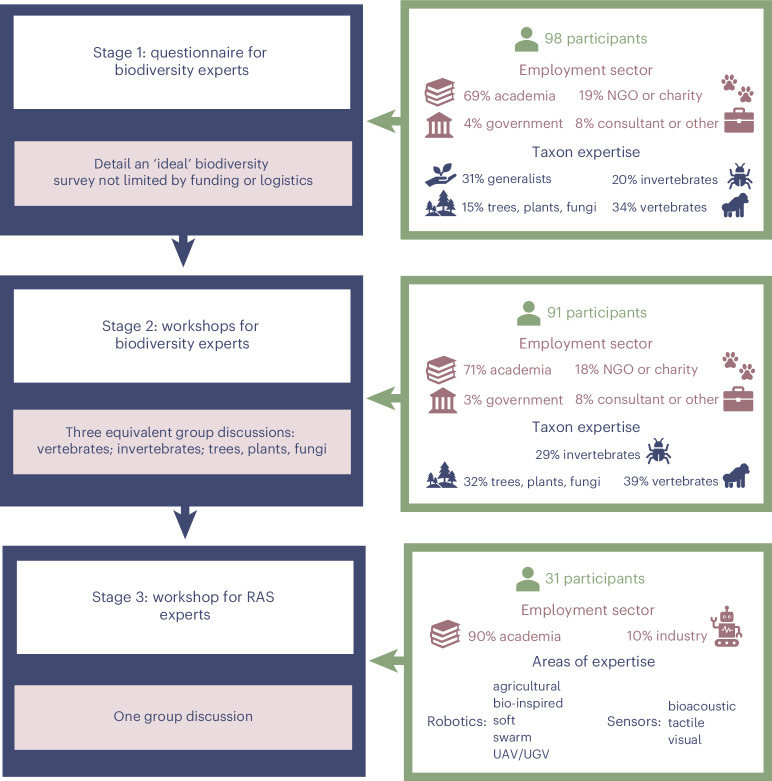
The modified Delphi technique used to identify the methodological barriers that currently hinder terrestrial biodiversity monitoring and the opportunities and challenges that RAS offer in overcoming these barriers. ‘Soft’, robots that use compliant materials to mimic natural locomotion; ‘swarm’, multiple robots, either homogeneous or heterogeneous, that are interconnected; UGV, uncrewed ground vehicle.

**Fig. 2:**
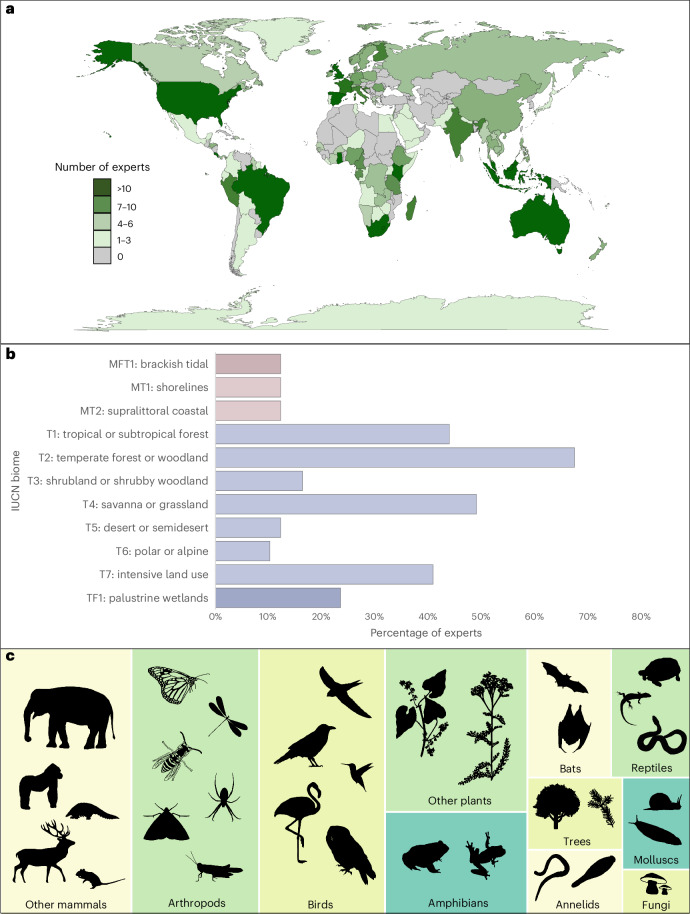
Terrestrial biodiversity monitoring experience of the 98 experts who completed stage 1 of the modified Delphi technique. **a**, Countries in which the experts had experience of conducting terrestrial biodiversity monitoring. **b**, Percentage of biodiversity experts with experience of monitoring biodiversity in each biome (86% of experts had conducted surveys in more than one biome), listed according to their IUCN classification^79^. MFT1, terrestrial transitional freshwater/marine; MT1–MT2, terrestrial transitional marine; T1–T7, terrestrial; TF1, terrestrial transitional freshwater. **c**, Relative numbers of experts with experience of monitoring each taxon (indicated by the area of each rectangle; 70% of experts had conducted surveys of more than one taxon). Bats are separated from other mammals, and trees from other plants, because the survey methods are notably different. Credits: World map outline in **a** is ©OpenStreetMap contributors; data are available under an Open Database License (https://openstreetmap.org/copyright). The icons in **c** are from PhyloPic (https://www.phylopic.org) contributed under a Creative Commons licence CC0 1.0.

**Extended Data Fig. 1:**
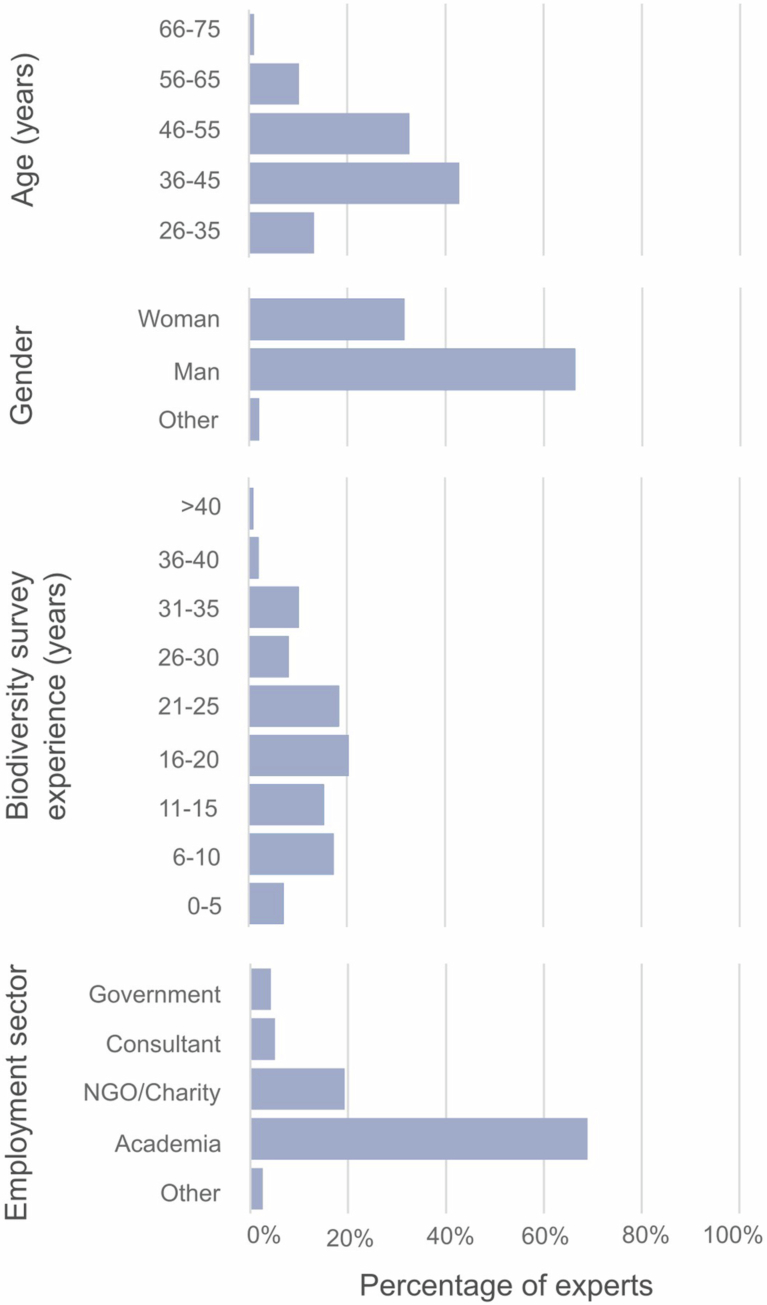
Socioeconomic/demographic background of the 98 biodiversity experts who completed Stage One of the modified Delphi technique. Percentage of experts according to their age, gender, biodiversity survey experience and employment sector. Eighteen experts were employed in more than one sector.

**Extended Data Fig. 2:**
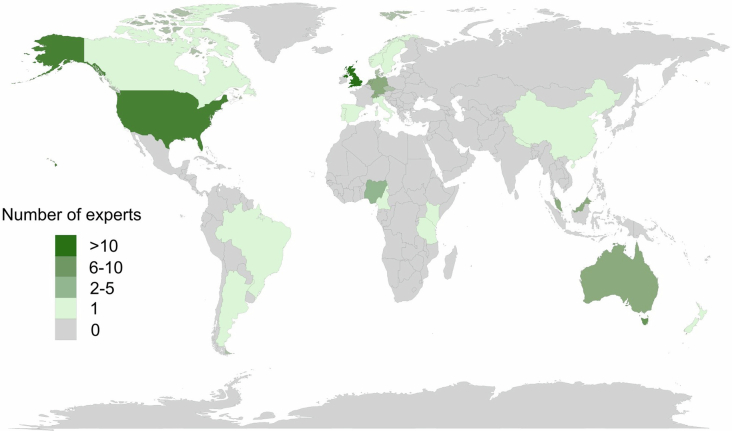
Countries in which the 98 biodiversity experts who completed stage 1 of the modified Delphi technique were resident. World map outline ©OpenStreetMap contributors, data available under the Open Database License openstreetmap.org/copyright.

Mobilizing RAS for biodiversity monitoring could substantially advance conservation efforts^13,14^. However, to date, there has been no systematic attempt to assess this potential. Here, we report the findings from a modified Delphi process^15^ that evaluated how we might adapt or develop current RAS to transform species surveys in terrestrial ecosystems (Fig. 1). Through an online questionnaire and workshops, we collated and synthesized knowledge from 98 biodiversity experts and 31 RAS experts, thereby identifying the major methodological barriers that currently hinder monitoring and exploring the opportunities and challenges that RAS offer in overcoming these barriers. The collective field survey experience of biodiversity experts encompassed 109 countries and a diversity of biomes and taxa (Fig. 2 and Extended Data Figs. 1 and 2).

## Results

**Fig. 3:**
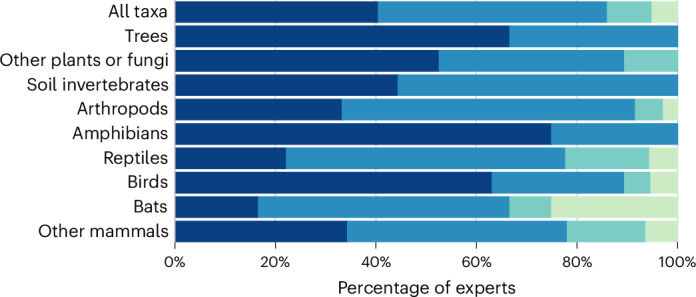
Categories of methodological barriers to monitoring terrestrial biodiversity, as reported by biodiversity experts during stage 1 of the modified Delphi technique. Percentages of biodiversity experts who identified methodological barriers, in four broad categories, that they expected to encounter in an ‘ideal’ survey that was not limited by funding or logistics, according to taxa. Dark blue, site access; blue, species and individual detection; teal, data handling and processing; light green, power and network availability.

In stage 1 of our modified Delphi technique, comprising an online questionnaire, we asked biodiversity experts to identify methodological barriers that they expected to encounter in an ‘ideal’ survey that was not limited by funding or logistics. We did not mention the use of RAS, or how they might be incorporated into surveys. Barriers fell into four broad categories: (1) site access, (2) species and individual detection, (3) data handling and processing, and (4) power and network availability (Table 1). The proportions of experts who highlighted barriers within each category varied by taxon; however, for all taxa, site access and species and/or individual detection were mentioned most frequently (Fig. 3).

**Table 1:** Methodological barriers that currently hinder terrestrial biodiversity monitoring and the opportunities and challenges that RAS offer in overcoming each of these barriers

Barrier category	Barrier	RAS opportunity or challenge	Brief description
1. Site access	Surveying over large spatial scales	Opportunity	Autonomous monitoring at landscape scales Replicating surveys at multiple sites over large geographical areas
	Surveying remote areas far from infrastructure	Opportunity	Accessing locations remote from roads and other infrastructure Monitoring sites that are time-consuming to access
	Surveying hazardous or inaccessible sites	Opportunity	Access to sites that need climbing (for instance, cliffs or forest canopies) Sampling sites at night or where personal safety or security is at risk
	Surveying taxa at random sites	Opportunity	Enabling representative sampling at suitable scale and stratification Avoiding sample pseudoreplication
	Surveying multiple locations simultaneously	Opportunity	Time-synchronous surveys at multiple sites Surveying taxa whose activity may be weather-dependent
	Surveying structurally complex habitats	Opportunity	Sampling within dense habitats (for instance, deadwood, grass tussocks or snow) Sampling soils, underground animal burrows, or bat colonies in caves or trees
	Surveying at high spatial resolution	Opportunity	Ability of sensor to get to exact locations repeatedly Enabling microscale tracking
	Designing environmentally robust sensors	Challenge	Resistance, resilience and durability of the sensors and/or probes in the field Being species-proof and avoiding risk of vandalism or theft
	Surveying restricted and off-limits locations	Challenge	Areas affected by legal, conflict and political issues Uncertainty of tenure or ownership status for many locations
2. Species and/or individual detection	Eliminating the need for multiple sensors	Challenge	Integration of multiple sensor types Ablility to deal with wide range of species sizes
	Discriminating or identifying individuals at distance	Challenge	Distance limitations of visual sensors (for instance, detection of plant ligules) Difficulties in identifying individuals of a species
	Surveying without disturbing taxa or habitats	Challenge	Non-invasive sensors that will not disturb species or habitats Impacts on non-target species
	Surveying through objects or in low light levels	Challenge	Detection when visibility is restricted (for instance, through vegetation or cloud) Detection of ectotherms at night
	Surveying ecological processes	Challenge	Monitoring interactions (for instance, pollination) or ecological processes Monitoring plant physiology
3. Data handling and processing	Handling high data volumes	Opportunity	Storage, energy costs and edge processing of extreme volumes of data Data transfer in real time to avoid data loss through sensor disturbance
	Identification of species in real time	Opportunity	Automated species identification by the RAS equipment Overcoming geographic and taxonomic bias
	Surveying over long temporal periods	Opportunity	Surveying sites continuously over extended periods Resurveying sites many times during a year and over many years
	Surveying rare, elusive or cryptic species	Challenge	Ensuring species detection (for instance, behaviourally cryptic diurnal taxa) Misidentifying rare or cryptic species and different sexes or life stages
	Surveying little-known or ‘difficult’ taxa	Challenge	Monitoring little-known taxa Monitoring species with poorly defined taxonomy
	Risk of misidentification by classifiers	Challenge	Identifying little-known or ’difficult’ taxa using AI tools Dealing with undescribed species
	Generating validated classifier training data	Challenge	Availability of training data for classifiers and/or expertise for validation Ground-truthing and geographical relevance of classifier data
	Designing RAS for non-expert operation	Challenge	Sensor easy to operate (for instance, to facilitate non-expert input) Accessibility of AI methods and training resources for non-experts
4. Power and network availability	Availability of communication network	Opportunity	Areas without access to mobile networks Network connections for real-time or cloud data access and storage
	Remote control and maintenance of RAS	Opportunity	Ability to control remotely (for instance, sensors in tree canopies) Self-reporting malfunctions for long-term sensor deployments
	Limited power availability	Challenge	Sustainable power, robust to climate, to support monitoring stations Reducing the weight of power systems
	Negative environmental impact of e-waste	Challenge	Environmental impact of production and/or decommissioning of RAS Retrieving inaccessible RAS equipment at end of life

These were identified by biodiversity experts during stage 2 of the modified Delphi technique.

**Fig. 4:**
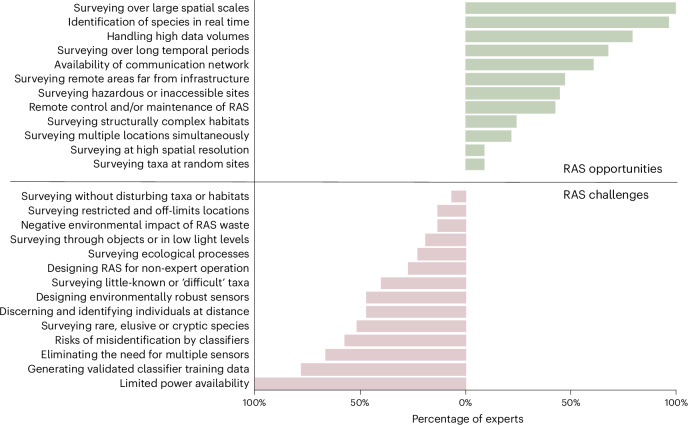
Opportunities and challenges associated with using RAS to monitor terrestrial biodiversity. These were identified by biodiversity experts during stage 2 of the modified Delphi technique. Each expert was allowed to select up to three opportunities and challenges that they believed would have the most profound impact on an ‘ideal’ survey.

In stage 2, which consisted of an online workshop, the same biodiversity experts considered the opportunities and challenges that RAS offered in terms of overcoming the barriers identified in stage 1 (Table 1 and Fig. 4). The opportunities identified most often involved the potential use of RAS to survey large spatial areas, with real-time species identification and handling of high data volumes. The major technological challenges highlighted with respect to RAS were power availability, generation of validated training data, elimination of the need for multiple sensor types and the risk of misidentification by automated classifiers^16^.

### Barrier category 1: site access

Biodiversity experts widely acknowledged issues pertaining to site access. All participants identified the potential of RAS to survey over large spatial scales as an opportunity. The ability of RAS to survey over large areas was also seen as facilitating ‘true habitat replicates to avoid pseudoreplication’. The opportunity to use RAS to survey repeatedly with high spatial resolution would bring a ‘level of confidence that the results are robust and repeatable’. Using multiple RAS to sample multiple sites simultaneously was viewed as ‘important for taxa whose activity may be especially weather-dependent’ (for instance, reptiles^17^). RAS surveys of areas distant from infrastructure would be beneficial where ‘lone working at remote locations is sometimes dangerous, especially where terrain is rugged’. Furthermore, RAS might transport heavy equipment to inaccessible areas.

In stage 3 of our modified Delphi technique, an online workshop, RAS experts proposed that biodiversity could be monitored using UAVs, uncrewed ground vehicles or legged (field) robots. Although UAVs are most commonly employed, legged robots with embedded intelligence have been used to monitor vegetation in dunes, screes, grasslands and forests^18^. A legged robotic system has also been shown to generate forest tree inventories faster and more cost-effectively than traditional forestry methods^19^. Recent locomotion developments are likely to extend the operational domain of legged robots to more complex ecosystems^20^. RAS could operate either independently, or collectively as ‘swarms’ (multiple robots, either homogeneous or heterogeneous, that are interconnected^7^). Multirobot swarms could operate by ‘coordinating activity and deciding when to sample, rather than just running on fixed schedules’. For example, multirobot swarms might use artificial intelligence (AI) to divide up a large area, ground-truth the habitat types and then sample from representative locations. UAVs could also place and retrieve sensors across an area using technology developed for environmental monitoring^21^. These methods may be suitable for surveying species such as snow leopards, *Panthera uncia*, which have very low population densities over extremely rugged terrain^22^.

RAS experts noted that RAS are extensively used to navigate through structurally complex areas (for instance, nuclear facilities^23^ or inside aircraft wings^24^). Tactile feedback manipulators^25^ could enable robots to move through dense scrub by ‘feeling’ their way, whereas UAVs could use visual navigation^26^ to avoid collisions within cluttered forests. Tree-climbing robots^27^, rather than humans, could survey forest canopies, which would ‘circumvent enormous training and h[ealth] & s[afety] issues’. Other technologies, including subcentimetre-sized soft crawling biomedical engineering robots^28^, may enable the monitoring of annelids in topsoil. A benefit of soft-bodied robots is their ability to flexibly change shape; they are also considered to be safer in environments where they might interact with humans or species^29^. Harsh weather conditions are challenging for researchers undertaking surveys. They also pose problems for RAS that can fail in extreme temperatures, humidity, rain, electrical storms and strong winds. RAS experts confirmed that ‘most lab[oratory]-built robots do not have great corrosion resistance’ and that commercial ‘electronic components are not built for arctic temperatures’. However, recently engineered ‘thermally agnostic’ drones, capable of working in very hot and cold environments, offer a potential way forward^30^.

Biodiversity experts commented that monitoring sites may be difficult to access for many reasons, including political and security issues or uncertainties surrounding ownership. Certain types of RAS (for instance, UAVs) also have military or surveillance connotations^31^. Illustrating this point, one expert reported that efforts to monitor biodiversity ‘had been met with fierce local resistance, with their drones routinely targeted by firearms’. The importance of working within legal constraints, engaging with local communities, and integrating RAS-collected data with local and indigenous knowledge of the environment^32^ was stressed.

### Barrier category 2: species and individual detection

To monitor terrestrial biodiversity effectively using visual cues, RAS sensors must be able to detect species over a wide size range (for instance, invertebrates from <<1 mm to 1 m (ref. ^33^) and vascular plants from ~1 cm to ~100 m (refs. ^34,35^)). The microscopic size of critical features is problematic for plant surveys, as identifying some species is dependent on almost-invisible ligules and hairs^36^. Similar difficulties are faced with invertebrates as it is ‘impossible to ID [identify] some taxa without dissection’. This places substantial demands on sensor design. Many biodiversity experts doubted whether the need for many sensors for multiple taxa^37^ could be eliminated. RAS experts agreed that ‘realistically, [RAS need to] use multiple sensors for different scales’. Some techniques that are being adopted in biodiversity monitoring might be further developed to extend sensor capabilities. For example, passive acoustic recordings could be enhanced through time-series analysis^38^ to address sound attenuation that hampers detection of quiet species. Chemosensors (‘electronic noses’), which are used in diverse agricultural and forestry applications^39^, might detect unique volatile organic compounds emitted by plants. Collection and removal of physical samples is also possible. Of particular interest are DNA fragments left behind by organisms in their environment (eDNA^40^) that can be used to detect the presence of species. Recent advances in the robotic collection of eDNA samples (for instance, from tree canopies^41^) offer great potential. However, monitoring biodiversity using eDNA requires further development to overcome limitations such as biases^42^ and the relationship between DNA biomass and abundance estimators^43^.

Using RAS to monitor cryptic species where visibility is restricted (for instance, in dense vegetation or low light) poses additional problems for sensors. The utility of RAS is also affected by the thermoregulation mechanism of target taxa. Passive infrared detectors are widely used for endotherms, but other methods are required for ectotherms, such as bioacoustics^44^ and image motion analysis^45^. Although flying UAVs generate sounds that may mask animal vocalizations, UAV-borne recorders have successfully recorded birds^46^ and bats^47^. As RAS technology continues to develop quieter platforms, the use of UAVs in bioacoustic monitoring is likely to increase.

The potential for RAS to also monitor ecological processes such as predation and decomposition was perceived as important, with biodiversity experts reflecting that ‘ecological function is about processes’ and that ‘it’s not the abundance of a tree species or a seed disperser species that matters, but whether the tree species is regenerating’. RAS experts confirmed that this would be difficult to achieve but pointed to recent successes in the use of RAS to monitor pollination, albeit in a simplified system^48^, and remote sensing of plant photosynthesis and primary productivity^49^.

Biodiversity experts recognized the challenge of performing RAS surveys while minimizing disturbance of species and habitats^31^. In the case of UAV-based surveys, disturbance of species can be caused by the shape of the UAV and its approach distance, airspeed, and flight pattern, as well as pilot competence and noise^50^. However, it was acknowledged that surveys by humans also cause disturbance^51^ and that ‘[there are] likely to be pros and cons for disturbance from humans versus robots’. RAS experts agreed that ‘aerial vehicles are noisy and many wheeled terrestrial vehicles can be destructive in terms of trampling’ but noted that the key to developing solutions lies in defining the criteria and thresholds for no or low disturbance to species or habitats^50^.

### Barrier category 3: data handling and processing

Ecologists often need to survey biodiversity over many days, months or years, rapidly generating large data volumes^52^. One biodiversity expert stated that ‘storage for extreme volumes of data is a top priority in the bioacoustic monitoring field.’ RAS experts highlighted several technologies that could help. The most commonly used method is edge processing, in which AI computations are used to preprocess data and reduce storage and data transmission requirements^53^. Other suggestions included AI prioritization of data storage based on sampling variation, using lossless data compression techniques^54^, and optimizing data storage using a wireless sensor network^55^ or data-mule drones^56^ to offload data from sensors. Alternatively, data transmission to cloud storage for subsequent offline processing would be possible if RAS could access a communication network. RAS experts emphasized good preparation before sampling so only relevant data are collected.

Recent advances in sound- and image-based biodiversity identification app technology (for instance, https://merlin.allaboutbirds.org, https://plantnet.org) were noted by several biodiversity experts. Virtually all biodiversity experts thought real-time species identification would be an opportunity associated with RAS but also recognized major challenges associated with automated identification. For example, three-quarters (Fig. 4) highlighted the lack of classifier training data and expert validation for most taxa, and more than half foresaw potential risks arising from species misidentification^57^. Additional concerns were raised regarding the accessibility of machine learning methods for non-experts, data ownership, lack of open-access data and data protection.

For some taxa, declining numbers of taxonomists will hamper the verification of species’ training data^58^. One biodiversity expert stated ‘you can’t replace the value of expert interpretation of species, management, habitats, and context’. However, others countered this saying that expert opinions can be fallible^59^. Indeed, a prerequisite of automated analysis is a huge library of expert-certified species images (or sounds), and ‘classifiers need to be trained with samples that are geographically relevant’. Some biodiversity experts expressed doubts about compiling suitable datasets, as ‘training data tend to be biased towards well sampled areas/groups’. This poses a particular problem for rare, elusive and cryptic species, which was seen as a challenge for RAS to address. Moreover, many biodiversity experts expressed doubts over monitoring little-known and ‘difficult’ taxa, emphasizing that classifiers ‘must be able to recognize when the species is unknown. For instance, not within its training set’. Another apprehension was that classifier identification errors might lead to threatened species being given incorrect IUCN conservation status^60^. It was therefore seen as important that human experts should evaluate error rates of AI species identification, with a consequent need to store raw data for independent validation.

There are few solutions currently available to overcome the difficulties associated with compiling huge annotated datasets for automated species identification. One technique suggested by RAS experts was the use of machine learning approaches that employ techniques with reduced data requirements, such as ‘few-shot learning’^61^. For instance, limited real data, supplemented by data augmentation^62^ with simulated data, could be used to identify large mammal species in camera trap images^63^. However, few-shot learning techniques applied without adequate validation can lead to serious misrepresentations of biodiversity^64^.

### Barrier category 4: power and network availability

Power availability was recognized by all biodiversity experts as a major issue related to monitoring of terrestrial species. They also remarked that some of the RAS challenges were interlinked. For example, whereas edge processing of sensor data and communication network capability could minimize data storage, it may increase RAS power requirements. The ability to control and maintain RAS equipment remotely was identified as an opportunity by biodiversity experts. Maintaining sensors is ‘very challenging, even in urban environments’, and surveyors ‘often need direct access for maintenance [and] signal proximity to control software’. RAS experts noted that this capability could be provided, but that ‘networks lack traceability of where the issues arise’, and that ‘the internet of things is not that mature’.

Although battery technologies have advanced rapidly, battery-powered robots and sensors generally have short operational lives before needing to recharge. Biodiversity experts undertaking monitoring during the winter observed that ‘cold can drain power sources very, very quickly’. Batteries carried by RAS need to power the robotic movement, the sensor(s) and the controller with storage memory. As a result, the endurance of hovering UAVs is typically 20–40 min (ref. ^65^). The use of solar power was thought to be helpful by biodiversity experts, but they noted that it ‘isn’t great for high latitude winters’, and ‘solar is not viable for [the] understorey’. RAS experts identified several currently available technologies that may help address the challenge of powering RAS. Efficient energy consumption has been demonstrated in multimodal robots that combine aerial and terrestrial locomotion modes within one platform^66^. A similar approach has been adopted in a solar-powered robot that minimizes energy consumption in the manner of tree sloths by traversing wires slowly while performing long-term environmental monitoring^67^. RAS experts also suggested that sensors could use low-powered microchips for onboard computing and energy-efficient cameras to reduce energy needs. Another method would be to employ homing robotic systems that return to recharging hubs to prolong operating times^68^. Other possibilities for providing sustainable power include microbial fuel cells^69^, harnessing rain^70^, triboelectric nanogenerators for mechanical energy harvesting^71^, thermoelectric energy harvesting from soil^72^ and chemical energy^73^. Addressing environmental impact is complex, but RAS experts stated that rapid progress is being made in developing biodegradable batteries^74^, sensors and soft robotic systems^75^.

Overall, the assessment of biodiversity and RAS experts was that widespread adoption of RAS for monitoring biodiversity requires further technological development, and that some areas are likely to be addressed relatively easily, whereas others pose greater challenges (Table 2).

**Table 2:**
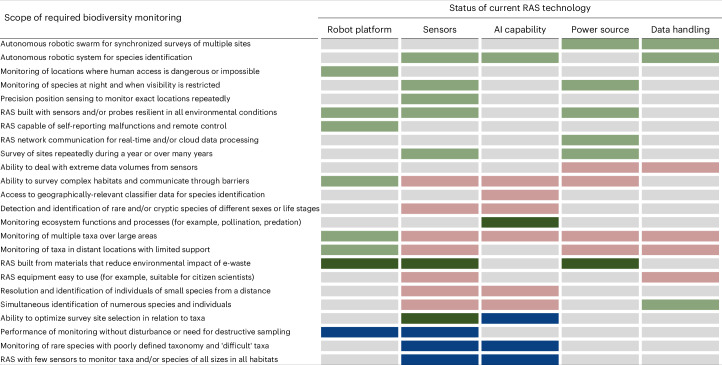
Status of current RAS technology available for terrestrial biodiversity monitoring

Coloured shading indicates the main areas in which technological developments are needed to enable RAS to perform each monitoring task: grey, field-tested technology exists; sage green, technology exists, but substantial limitations need to be overcome; dark green, working prototypes exist; pink, technology is still in research and development phase; blue, major technological breakthrough required.

## Discussion

For common species of some taxa, including birds and mammals, RAS are already providing valuable survey data, and this capability is increasing. For these taxa, the main limitation is accurate identification of lesser-known species, for which geographically relevant classifier data may be lacking. In ‘difficult’ taxa such as fungi, this constraint poses a severe problem. However, the lack of classifier data is only one factor impeding the utility of RAS for biodiversity monitoring. This is because of the complex interrelationships between sensors and sensing techniques used to detect species; the management, communication and processing of sensor data; and the provision of power for these tasks, as well as for the robotic platform.

Many of the enabling technologies and capabilities needed for RAS to monitor terrestrial biodiversity effectively already exist, although they have often been developed for different applications^8–12,23–30^. Several types of robotic platform are already used in biodiversity surveys^11^, and rapid development progress (for instance, for subterranean access^28,29^) suggests that this will not be the primary bottleneck. The critical limitations to overcome are sensors and sensing techniques^36–49^ with classifier databases^57–60^, where major breakthroughs are needed. Progress in these areas could rapidly advance accurate species identification across more taxa but will be dependent on new methods of processing large data volumes^52–54,61–64^ in real time. Although not an immediate constraint, power source developments will become increasingly critical to sustain RAS autonomy as the capabilities of other components advance. Without enhanced power availability, RAS can only be deployed to monitor biodiversity for short time periods in some ecosystems and geographical regions. It is not possible to predict when such transformative breakthroughs may occur, but recent advances in power source technology^69–74^ are cause for optimism.

Adapting RAS to new environments might be problematic, as considerable time and resources are required to create, service and support robust systems suited to working in uncontrolled conditions. Field-testing of RAS as fully integrated units for terrestrial biodiversity monitoring is a critical step in defining the boundaries of their capabilities. Given these constraints, it may initially be more efficient to deploy multiple stationary sensor systems rather than mobile RAS. This approach could provide the spatial coverage that mobile robots offer, while avoiding many challenges associated with developing robust navigation and power management systems. Alternatively, readily available RAS could be more widely deployed for repetitive monitoring of well-known taxa and easily accessible ecosystems^12–14^. This could free human surveyor time to focus on specific taxa, habitats and ecosystems for which RAS are currently underdeveloped.

Despite the challenges, the development of RAS able to track changes in species abundance and community composition could deliver profound advances in conservation. In the present study, most biodiversity experts foresaw many opportunities associated with RAS but viewed them as additional tools to supplement rather than supplant existing survey methods. There was some hesitation about the suitability of RAS for certain taxa (for instance, those for which genomic data are needed for accurate identification^4^). One overarching issue was that RAS could quickly generate huge volumes of biodiversity data that could be used to inform policy and practice without critical evaluation. It is unclear whether taxonomic bias, with a focus on some species to the detriment of others^76^, may increase or decrease with the use of RAS. Concerns were also raised regarding high costs, e-waste, ethical implications and diversion of resources from other conservation work. Nevertheless, RAS integrated into well-structured, goal-based programmes with standardized protocols could lead to major progress in monitoring of terrestrial biodiversity. As one biodiversity expert observed ‘if [RAS] could monitor just 10% of species reliably across all taxonomic groups at appropriate scales and resurvey intervals, it would be a substantial improvement on current approaches’.

Genuinely transdisciplinary approaches to terrestrial biodiversity monitoring need to be fostered between biodiversity and RAS experts, so that ideas and technologies can be codeveloped effectively. Biodiversity experts generally have limited formal training in RAS and big data. Likewise, RAS experts do not routinely consider the complexity of biodiversity, ecosystem functioning and the practicalities associated with field-based monitoring. By promoting and funding cross-disciplinary collaboration aimed at adapting RAS for conservation applications, governments, philanthropists and organizations can drive major progress. One such example is the ‘Natural Robotics Contest’ (https://www.naturalroboticscontest.com/), an environmental robot design competition. In the longer term, education strategies at all levels should seek to establish and augment interdisciplinary thinking among aspiring engineers, ecologists and computer scientists^77^. This could be achieved by highlighting the major methodological challenges and need for improved technology to support terrestrial biodiversity monitoring in undergraduate engineering and computer science courses, as well as providing explanations of cutting-edge technological applications in ecology and conservation courses. Future generations of researchers may then be able to communicate and work together more readily, bridging the traditional disciplinary boundaries between ecology and engineering.

## Methods

We undertook our modified Delphi technique, a method that is applied widely in conservation and environmental sciences^15^, between April and June 2023. The technique involves a structured and iterative survey of a group of participants that aims to capture a broad range and depth of contributions. It has several advantages over standard approaches to gathering opinions from groups of people. For example, participant contributions are anonymous, which minimizes potential biases resulting from social pressures such as groupthink, halo effects and the influence of dominant individuals^15^.

Our Delphi approach comprised three stages: an online questionnaire and online workshop for biodiversity experts, followed by an online workshop for RAS experts (Fig. 1). Participants were asked to provide informed consent before participating in any of the activities. We made them aware that their involvement was entirely voluntary, that they could stop at any point and withdraw from the process without explanation, and that the data they provided via the questionnaire and workshop would be anonymous and unidentifiable. Ethical approval was granted by the School of Anthropology and Conservation Research Ethics Committee at the University of Kent (reference 394 2023).

### Stage 1: biodiversity expert online questionnaire

We used a mixed approach to recruiting biodiversity experts for stage 1 to minimize the likelihood of bias associated with relying on a single method. By using global professional networks and identifying authors of recent papers on monitoring of terrestrial taxa, we identified 334 experts from across the world. We also found an additional 154 experts by contacting relevant research institutes, non-governmental organizations and conservation agencies, and by snowball sampling (invitees suggesting other biodiversity experts who might be interested in participating). Our aim was to recruit experts with experience of biodiversity surveys in a diverse range of biomes and covering all terrestrial taxa (Fig. 2 and Extended Data Figs. 1 and 2). Of the 488 biodiversity experts (35% women) in 43 countries who were invited, 98 experts (33% women) in 24 countries took part in stage 1.

The questionnaire was delivered using the online platform Qualtrics (https://qualtrics.com). We asked participants to list their country of residence; their employment sector; their experience of monitoring taxa, habitats, and ecosystems; and the countries in which they had conducted or facilitated terrestrial biodiversity monitoring. We asked participants to detail an ‘ideal’ biodiversity survey that was not limited by funding or logistics. We did not mention RAS and how it might be incorporated into surveys to ensure that participants were not influenced by their understanding of the capabilities and limitations of RAS. Participants were asked to specify which terrestrial taxa and ecosystems their monitoring would focus on and the methodological barriers that would need to be overcome to make the survey possible. We piloted and pretested the questionnaire content, which helped us to refine the wording of questions and definitions of terminology. We used an inductive approach to analyse the qualitative questionnaire responses. By synthesizing participant statements, we collated the data into four broad barriers (Fig. 3), which were the basis of discussion in stage 2.

### Stage 2: biodiversity expert online workshops

The same group of 98 biodiversity experts were invited to take part in an online workshop, organized on Teams, which aimed to assess the potential for RAS to resolve the barriers articulated in stage 1. Seven participants who had completed the questionnaire did not continue to stage 2. The remaining 91 participants (34% women) were allocated to one of three groups according to whether they self-identified as experts in surveying vertebrates (*n* = 36 participants); invertebrates (*n* = 26); or trees, plants and/or fungi (*n* = 29). Separate workshops were held for each group simultaneously, with each workshop following the same format.

Each workshop opened with a summary of the overall project and its aim, as well as a description of planned workshop activities. We presented the barriers in written format using Padlet (https://padlet.com/), a collaborative web platform where participants can access, upload and organize shared content. We asked participants to consider the opportunities and challenges that RAS offer with respect to overcoming these barriers within each of our four broad barriers (site access, species and individual detection, data handling and processing, and power and network availability; Table 1). We asked participants to clarify, expand, join or add new barriers wherever they felt necessary and to comment on the relevance and appropriateness of the RAS opportunities and/or challenges that emerged. Finally, for each of the four broad categories of barrier, we asked participants to select up to three RAS opportunities and challenges that they believed would have the most profound impact on their ‘ideal’ survey (Fig. 4).

### Stage 3: RAS expert online workshop

We used a mixed approach to recruit RAS experts to participate in our RAS online workshop. Our objective was to include global experts working at the forefront of RAS applications and development, including those working on closely related technologies such as sensors, AI and machine learning. Relevant experts were identified among authors of recent papers, from professional networks and mailing lists (for instance, the UK-RAS network), and by snowball sampling. Using this information, we emailed 196 experts (21% women) in 24 countries, inviting them to participate in an online workshop, organized on Teams, to discuss the applications of RAS to terrestrial biodiversity monitoring. A pool of 31 RAS experts (26% women) from eight countries took part. The smaller number of experts taking part in this workshop, compared with the biodiversity workshop, reflected the wide range of taxon, biome and global expertise we required from biodiversity specialists.

We began the workshop with an introduction to biodiversity, ecosystems and monitoring methods currently used to survey different taxa. This was followed by discussions of the barriers that had been identified by the biodiversity experts in stage 2. The barriers were grouped into the same four broad categories that had been used previously. RAS experts were asked to identify existing RAS capabilities that were directly transferable to a terrestrial biodiversity monitoring context, as well as nascent technologies or new ideas that might be relevant for the future. Again, we used an inductive approach to analyse the qualitative data derived from the workshop. This enabled us to determine existing RAS capabilities that are closely aligned with biodiversity monitoring requirements, how these capabilities could be extended and potential priorities for future RAS developments.

### Reporting summary

Further information on research design is available in the Nature Portfolio Reporting Summary linked to this article.

## Supplementary information

Reporting Summary

## Data Availability

The anonymized dataset generated and analysed during this study is available^78^ via the University of Kent Data Repository at 10.22024/UniKent/01.01.546.
